# Inferring Time-Varying Network Topologies from Gene Expression Data

**DOI:** 10.1155/2007/51947

**Published:** 2007-04-12

**Authors:** Arvind Rao, Alfred O Hero, David J States, James Douglas Engel

**Affiliations:** 1 Department of Electrical Engineering and Computer Science, University of Michigan, Ann Arbor, MI 48109-2122, USA; 2 Bioinformatics Graduate Program, Center for Computational Medicine and Biology, School of Medicine, University of Michigan, Ann Arbor, MI 48109-2218, USA; 3 Department of Human Genetics, School of Medicine, University of Michigan, Ann Arbor, MI 48109-0618, USA; 4 Department of Cell and Developmental Biology, School of Medicine, University of Michigan, Ann Arbor, MI 48109-2200, USA

## Abstract

Most current methods for gene regulatory network identification lead to the inference of steady-state networks, that is, networks prevalent over all times, a hypothesis which has been challenged. There has been a need to infer and represent networks in a dynamic, that is, time-varying fashion, in order to account for different cellular states affecting the interactions amongst genes. In this work, we present an approach, *regime-SSM*, to understand gene regulatory networks within such a dynamic setting. The approach uses a clustering method based on these underlying dynamics, followed by system identification using a state-space model for each learnt cluster—to infer a network adjacency matrix. We finally indicate our results on the mouse embryonic kidney dataset as well as the T-cell activation-based expression dataset and demonstrate conformity with reported experimental evidence.

## 

[1,2,3,4,5,6,7,8,9,10,11,12,13,14,15,16,17,18,19,20,21,22,23,24,25,26,27,28,29,30,31,32,33,34,35,36]
